# Budget Impact of RefluxStop™ as a Treatment for Patients with Refractory Gastro-oesophageal Reflux Disease in the United Kingdom

**DOI:** 10.36469/001c.90924

**Published:** 2024-01-11

**Authors:** Sam Harper, Lukasz Grodzicki, Stuart Mealing, Elizabeth Gemmill, Paul Goldsmith, Ahmed Ahmed

**Affiliations:** 1 York Health Economics Consortium, York, UK; 2 Sherwood Forest Hospitals NHS Foundation Trust, Nottingham, UK; 3 Central Manchester University Hospital NHS Foundation Trust, Manchester, UK; 4 Department of Surgery and Cancer Imperial College London, London, UK

**Keywords:** RefluxStop™, budget impact, NHS, proton pump inhibitor, laparoscopic Nissen fundoplication, magnetic sphincter augmentation, gastro-oesophageal reflux disease

## Abstract

**Background:** Gastro-oesophageal reflux disease (GORD) is a common condition associated with heartburn and regurgitation. Standard of care for GORD patients in the UK involves initial treatment with proton pump inhibitors (PPIs) and laparoscopic antireflux surgery in patients unwilling to continue or intolerant of long-term PPI treatment. Recently, RefluxStop™, a novel, implantable medical device, has proven to be an efficacious and cost-effective treatment for patients with GORD. The current analysis aimed to describe the budget impact of introducing RefluxStop™ within National Health Service (NHS) England and Wales.

**Objectives:** To estimate the more immediate, short-term clinical and economic effects of introducing RefluxStop™ as a therapeutic option for patients with GORD treated within NHS England and Wales.

**Methods:** A model adherent to international best practice guidelines was developed to estimate the budget impact of introducing RefluxStop™ over a 5-year time horizon, from an NHS perspective. Two hypothetical scenarios were considered, one without RefluxStop™ (comprising PPI treatment, laparoscopic Nissen fundoplication, and magnetic sphincter augmentation using the LINX® system) and one with RefluxStop™ (adding RefluxStop™ to the aforementioned treatment options). Clinical benefits and costs associated with each intervention were included in the analysis.

**Results:** Over 5 years, introducing RefluxStop™ allowed the avoidance of 347 surgical failures, 39 reoperations, and 239 endoscopic esophageal dilations. The financial impact of introducing RefluxStop™ was £3 029 702 in year 5, corresponding to a 1.68% increase in annual NHS spending on GORD treatment in England and Wales.

**Discussion:** While the time horizon was too short to capture some of the adverse events of PPIs and complications of GORD, such as the development of Barrett’s esophagus or esophageal cancer, the use of RefluxStop™ was associated with a substantial reduction in surgical complications, including surgical failures, reoperations, and endoscopic esophageal dilations. This favorable clinical profile resulted in cost offsets for the NHS and contributed to the marginal budget impact of RefluxStop™ estimated in the current analysis.

**Conclusions:** Introducing RefluxStop™ as a treatment option for patients with GORD in England and Wales may be associated with clinical benefits at the expense of a marginal budget impact on the NHS.

## INTRODUCTION

Gastro-oesophageal reflux disease (GORD) is a common, chronic gastrointestinal condition in which gastric contents flow back into the oesophagus, resulting in symptoms of heartburn, regurgitation, bloating, excessive salivation, and impaired sleep.^1^ GORD also increases the risk of developing the precancerous condition Barrett’s oesophagus and oesophageal cancer.^2^ Nearly 10% of the UK population are estimated to be affected by GORD.^2^

Currently, the National Institute for Health and Care Excellence (NICE) recommends that patients treated within the National Health Service (NHS) receive proton pump inhibitors (PPI) as initial treatment for GORD. Surgical management (ie, a laparoscopic Nissen fundoplication or magnetic sphincter augmentation [MSA]) using the LINX^®^ system may be offered to patients who are intolerant to or unwilling to accept prolonged treatment with PPIs.^3,4^ During a laparoscopic Nissen fundoplication procedure, the top of the stomach is folded and stitched to reduce the size of the opening between the stomach and oesophagus.^3^ Similarly, MSA with the LINX^®^ system also aims to reduce the size of this opening by inserting a ring of magnetic beads around the oesophagus, just above the stomach.^4^

In light of the limited treatment options comprising the standard of care within the NHS in England and Wales, recent years have brought potential advances in the treatment of GORD. MSA using the LINX^®^ system has been used in patients who do not respond well to treatment with PPIs^5,6^ and in January 2023 was recommended by NICE as part of routine care.^4^ Recently, a novel implantable, nonactive, single-use device (RefluxStop™, Implantica, Zug, Switzerland), which restores normal oesophageal anatomy and function without affecting the passage of food, has been developed as a treatment for patients with GORD who are eligible for laparoscopic surgery. The RefluxStop™ procedure involves laparoscopic surgery where the device is implanted on the outer stomach wall.^7^ RefluxStop™ received a CE mark across the European Union (then including the United Kingdom) in August 2018, allowing for RefluxStop™ to be marketed and sold in these countries for the treatment of acid reflux.^8^ CE mark approval was based on the positive results of a prospective, single-arm, multicenter study enrolling 50 patients with chronic GORD who required daily PPI treatment.^7^ This study demonstrated that RefluxStop™ was a safe, well-tolerated treatment for GORD, with efficacy that may improve upon the current standard of care.^7^ The study met its primary efficacy endpoint; at 1 year post-surgery, patients implanted with RefluxStop™ experienced on average 86% improvement in GORD-related symptoms measured using a well-established, disease-specific questionnaire (GORD–Health-Related Quality of Life [GORD-HRQL]^9^).^7^ Laparoscopic implantation of RefluxStop™ also resulted in a decline in objectively measured GORD severity; at 6 months post-surgery, 98% of the patients displayed normal oesophageal pH on 24-hour monitoring, meeting a secondary outcome of the study.^7^ The safety profile of RefluxStop™ in the CE mark trial was consistent with laparoscopic surgery, as no serious adverse events relating to the RefluxStop™ device were reported and any complications were related to laparoscopic surgery rather than the RefluxStop™ device.^7^

A recent health-economic evaluation demonstrated the cost-effectiveness of RefluxStop™ when assessed over a patients’ lifetime horizon from the perspective of the NHS in England and Wales.^10^ The objective of the current study was to estimate the more immediate, short-term clinical and economic effects of introducing RefluxStop™ as a therapeutic option for patients with GORD treated within NHS England and Wales.

## METHODS

### Model Overview

A budget impact model adherent to the recommendations of the International Society for Pharmacoeconomics and Outcomes Research (ISPOR)^11^ was developed. The model utilized a 1-year cycle length and estimated the budget impact of introducing RefluxStop™ over a 5-year time horizon from the perspective of the NHS in England and Wales. Two hypothetical scenarios were considered: one with existing interventions (PPI-based medical management, laparoscopic Nissen fundoplication, and MSA using the LINX^®^ system) but without RefluxStop™, and another in which RefluxStop™ was introduced in addition to the currently existing interventions.

**Supplementary Figure 1** presents the model schematic. First, the population potentially eligible for treatment with RefluxStop™ (ie, patients diagnosed with GORD who currently take PPI medication and/or who have not had previous surgery and are eligible for/willing to undergo surgery) and the change in the size of this population over the 5-year model horizon were identified. The population was consistent with those who participated in the RefluxStop™ CE mark trial. Market shares of the available treatment options were then applied to the eligible population, and costs specific to each intervention (including the cost savings arising from the benefits of each technology, ie, cost offsets) were evaluated to obtain the total costs associated with the scenario with RefluxStop™ and those without RefluxStop™. The difference between these two scenarios represents the net budget impact of introducing RefluxStop™.

### Modeled Population

The modeled population included all those who may potentially benefit from the introduction of RefluxStop™, namely, patients with GORD who receive PPI medication or who have not had previous surgical treatment for GORD and are eligible and willing to undergo surgery. The model considered both prevalent patients with GORD and incident cases (ie, patients newly developing GORD during the model time horizon).

Estimates of population size and its projected growth in England and Wales were obtained from the Office for National Statistics (ONS) database.^12,13^ GORD prevalence and incidence rates in the UK general population, and the annual incidence of laparoscopic antireflux surgery were obtained from the literature. Prevalence of GORD (14.5%) and annual incidence of new GORD cases (0.5%) were based on systematic reviews of GORD epidemiology.^14,15^ The proportion of GORD patients treated within the NHS (6.6%) was derived from a pragmatic analysis of healthcare cost of managing GORD in the Dorset Clinical Commissioning Group, extrapolated to the entire NHS England and Wales.^16^ The number of antireflux surgeries among patients with GORD was 4.9 per 100 000 persons per year, based on a review of laparoscopic antireflux surgery in England.^17^

### Market Shares

Market shares of PPI-based medical management, laparoscopic Nissen fundoplication, and MSA, as well as the projected market share of RefluxStop™, were obtained from market research conducted by RefluxStop’s manufacturer, Implantica, and from expert opinion. The market shares are presented in **Table 1** for both the scenario without RefluxStop™ and the scenario with RefluxStop™. To accommodate for the uncertainty associated with real-world uptake of RefluxStop™, 2 additional scenarios were also considered. In one scenario, the rate of RefluxStop™ uptake was halved, while in the other scenario, the rate was doubled. The corresponding market shares in each model year are presented in **Table 1**.

**Table 1.** Market Share of Interventions Considered in the Scenarios With and Without RefluxStop™

**Treatment**	**Market Share (%)**				
	**Year 1**	**Year 2**	**Year 3**	**Year 4**	**Year 5**
Scenario without RefluxStop™ (all scenarios)					
Nissen fundoplication	89.7	79.6	69.6	63.0	56.4
MSA	10.3	20.4	30.4	37.0	43.6
Base case scenario with RefluxStop™					
RefluxStop™	5.1	10.2	15.2	18.5	21.8
Nissen fundoplication	84.6	69.4	54.4	44.5	34.7
MSA	10.3	20.4	30.4	37.0	43.6
Scenario with RefluxStop™ (reduced rate of RefluxStop™ uptake)					
RefluxStop™	2.6	5.1	7.6	9.3	10.9
Nissen fundoplication	87.2	74.5	62.0	53.7	45.6
MSA	10.3	20.4	30.4	37.0	43.6
Scenario with RefluxStop™ (increased rate of RefluxStop™ uptake)					
RefluxStop™	10.3	20.4	30.4	37.0	43.6
Nissen fundoplication	79.5	59.2	39.1	26.0	12.9
MSA	10.3	20.4	30.4	37.0	43.6

Abbreviation: MSA, magnetic sphincter augmentation.

For the model calculations, the market share values were multiplied by the population size to estimate the number of people receiving each of the treatment options over the 5 years. Due to the surgical nature of most of the treatments considered in the model, it was assumed that those people who had the procedure previously would not switch to another treatment. Treatment switching in patients who underwent surgery after initially receiving PPI-based medical management was included in the per-patient cost estimates. Therefore, the population entering the model included all prevalent GORD patients in Year 1, and only incident cases in Years 2-5.

### Costs

The cost per patient per year for each treatment reflected all costs associated with each treatment option and was obtained from the previously published cost-effectiveness model of RefluxStop™, which also applied the perspective of NHS England and Wales.^10^ The methodology of the cost-effectiveness model has been described previously.^10^ Briefly, the major cost categories captured were (1) treatment costs, comprising the costs of PPI medications and surgical treatments, with the latter including procedure costs and, for MSA and RefluxStop™ only, device and training costs; (2) the costs of diagnosing and treating Barrett’s oesophagus and oesophageal cancer in patients who developed the conditions; (3) the costs of managing adverse events associated with PPIs (chronic kidney disease,^18^ cardiovascular events,^19^ fractures,^20^ pneumonia,^21^ *Clostridium difficile* infections,^22^ and stomach cancer^23^) and the costs of managing adverse events associated with surgical treatment (conversion from laparoscopic to open surgery, oesophageal dilation, additional surgery for major complications and, for RefluxStop™ and MSA only, device removal). To ensure the costs used in the model were as relevant to NHS England as possible, unit costs were sourced from NHS schedule costs 2019/20^24^ whenever possible. Where data are used from older sources in the literature, the values were inflated using the Personal Social Services Research Unit (PSSRU) inflation indices.^25^

The total annual per-patient costs associated with each modeled treatment are presented in **Table 2**. The costs in year 1 were higher for surgical treatment options than for medical management, as they included the surgery and device costs. In contrast, the costs incurred during years 2-5 with the surgical treatment options were mainly related to follow-up, and therefore lower than for medical management.

**Table 2.** Cost per Patient per Year for the Different Treatments Assessed

**Treatment**	**Per-patient Cost (£)**					
	**Year 1**	**Year 2**	**Year 3**	**Year 4**	**Year 5**	**Total (Years 1-5)**
Medical management	286	292	275	260	246	1359
RefluxStop™	10 489	45	45	48	50	10 677
Nissen fundoplication	5749	86	77	80	81	6073
MSA	8856	134	114	116	117	9337

Abbreviation: MSA, magnetic sphincter augmentation.

### Analysis

The total budget required for each treatment option was determined by multiplying the number of people receiving each treatment by the cost per specific year associated with that treatment option, so that patients in year 2 of their treatment would receive the year 2 cost, those in year 3 of their treatment would receive year 3 costs, etc. The total costs for each year were then summarized over the 5-year time horizon to generate the final healthcare system budget required for the scenarios with and without RefluxStop™. The net budget impact of introducing RefluxStop™ was calculated as the difference between the budget required to fund the scenario with RefluxStop™ and that without RefluxStop™. Scenario analysis were used to test the robustness of the model to changes in market uptake rates (the key driver of budget impact).

## RESULTS

The number of patients who receive RefluxStop™ was estimated at 150 in the first year of its availability and increased to 650 in the fifth year, with a corresponding decline in the annual number of patients who undergo Nissen fundoplication, from 2626 in year 1 to 1685 in year 5 (Table 3).

**Table 3.** Number of Patients Receiving Each Treatment per Model Year in Base Case Scenarios With and Without RefluxStop™

**Modeled Year**	**Patients Receiving Treatment (n)**						
	**Scenario Without RefluxStop™**			**Scenario With RefluxStop™**			
	**PPI-Based Medical Management**	**Nissen Fundoplication**	**MSA**	**PPI-Based Medical Management**	**Nissen Fundoplication**	**MSA**	**RefluxStop™**
1	569 777	2626	300	569 777	2626	300	150
2	16 873	2342	600	16 873	2342	600	300
3	16 851	2057	900	16 851	2057	900	450
4	16 831	1872	1100	16 831	1872	1100	550
5	16 811	1685	1300	16 811	1685	1300	650

Abbreviations: MSA, magnetic sphincter augmentation; PPI, proton pump inhibitor.

**Figure 1.**
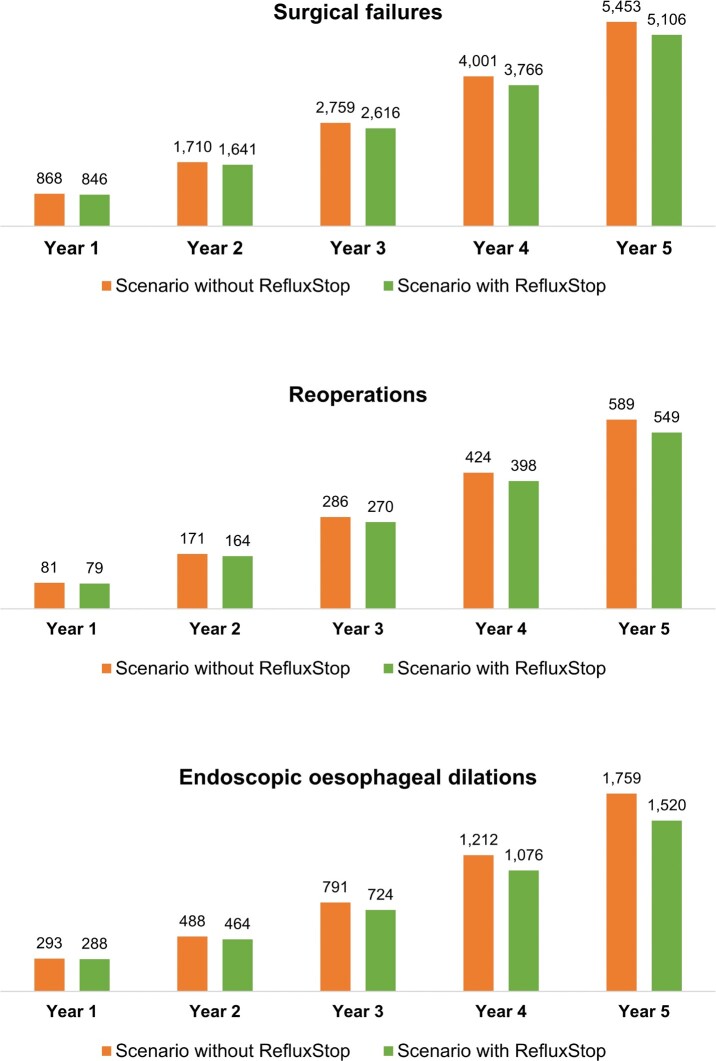
Clinical Outcomes of Base Case Scenarios With and Without RefluxStop™ Over the 5-Year Model Time Horizon

Over the model’s 5-year time horizon, introduction of RefluxStop™ was associated with improved clinical outcomes, with 347 surgical failures, 39 reoperations, and 239 endoscopic oesophageal dilations avoided (representing 6.8%, 7.2%, and 15.7% reduction, respectively) relative to the scenario without RefluxStop™ (**Figure 1**).

**Figure 2.**
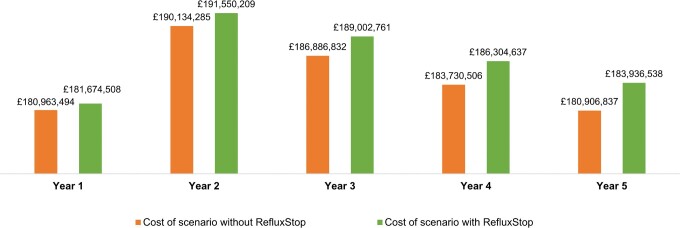
Total Costs of Base Case Scenarios With and Without RefluxStop™

The total costs over the 5-year model time horizon associated with the scenarios including and excluding RefluxStop™ are presented in **Figure 2**. The net 1-year, 3-year, and 5-year budget impact of introducing RefluxStop™ was £711 014, £2 115 929, and £3 029 702 per year, respectively, which corresponded to 0.39%, 1.14%, and 1.68% increases per year in overall NHS expenditure for GORD treatment in England and Wales (**Table 4**).

**Table 4.** Total Costs Estimated in Base Case Scenarios With and Without RefluxStop™ and Budget Impact

	**Year 1**	**Year 2**	**Year 3**	**Year 4**	**Year 5**
Eligible population (n)	572 703	592 519	612 327	632 130	651 926
Population expected to receive RefluxStop™ (n)	150	450	900	1450	2100
Cost of treatment pathway without RefluxStop™ (£)	180 963 494	190 134 285	186 886 832	183 730 506	180 906 837
Cost of treatment pathway with RefluxStop™ (£)	181 674 508	191 550 209	189 002 761	186 304 637	183 936 538
Nominal net budget impact (£)	711 014	1 415 924	2 115 929	2 574 132	3 029 702
Net budget impact as percentage of NHS spending for GORD treatment (%)	0.39	0.75	1.13	1.40	1.68

Abbreviations: GORD, gastro-oesophageal reflux disease; NHS, National Health Service.

In the scenario in which the rate of uptake of RefluxStop™ was halved relative to the base case, the 5-year reductions in the number of surgical failures, reoperations, and endoscopic dilations were 174, 20, and 120, respectively. The 1-year budget impact of introducing RefluxStop™ in this scenario was £335 507, corresponding to an 0.20% increase in overall NHS expenditure for GORD treatment. The 5-year budget impact was £1 514 851, amounting to an 0.84% increase in NHS expenditure.

In the scenario exploring a doubled rate of RefluxStop™ uptake, the 5-year reductions in the number of surgical failures, reoperations and endoscopic dilations were 695, 79, and 479, respectively. The budget impact associated with increased uptake of RefluxStop™ in the UK was £1 422 029 over 1 year, which translated to an 0.79% increase in NHS spending on GORD treatment, and £6 059 403 over 5 years, translating to a 3.36% increase in spending.

## DISCUSSION

RefluxStop™ has demonstrated a favorable safety and efficacy profile in its pivotal CE mark study,^7^ and a recent analysis suggests it is highly likely to be a cost-effective treatment for GORD patients in England and Wales at the standard cost-effectiveness threshold of £20 000 per quality-adjusted life-year gained.^10^ The current analysis focused on the short-term health and economic impact of introducing RefluxStop™ as a treatment option on the NHS. While the time horizon of 5 years was too short to meaningfully capture some of the adverse events of PPIs and complications of GORD itself, such as the development of Barrett’s oesophagus or oesophageal cancer, the use of RefluxStop™ was associated with a substantial reduction in surgical complications, including surgical failures, reoperations, and endoscopic oesophageal dilations. This favorable clinical profile resulted in cost offsets for the NHS and contributed to the marginal budget impact of RefluxStop™ estimated in the current analysis.

PPIs and, in selected patients, laparoscopic Nissen fundoplication, are recommended in the NICE guideline for GORD management^3^ and form the mainstay of GORD treatment within the NHS. However, long-term PPI use is associated with a number of potential adverse events, including kidney disease, infections, and myocardial infarction, among others.^26^ Furthermore, up to a third of GORD patients do not achieve sufficient symptom relief with PPI treatment.^27^ With regard to laparoscopic Nissen fundoplication, although this type of surgery has been reported to provide superior control of GORD symptoms, it is also associated with an up to 5-fold increase in the risk of dysphagia compared with PPI treatment.^28^ Furthermore, by 5 to 10 years post-surgery, some patients experience a recurrence of burdensome GORD symptoms such as heartburn and regurgitation.^29^ Therefore, there remains a group of patients who do not achieve optimal control of GORD symptoms with standard of care treatments. A novel treatment modality, MSA using the LINX^®^ system, has recently been recommended by NICE as part of routine care,^4^ and its adoption is likely to be gradual. Consequently, there remains a need for additional treatment options for patients with GORD.

New treatments for GORD are likely to become even more important from the population health perspective over the coming years with the aging population of the UK. The Global Burden of Disease Study 2017 estimated that the number of cases of GORD as well as the absolute number of years lived with disability due to the condition increased in the UK between 1990 and 2017; however, the corresponding age-standardized rates did not increase, consistent with the higher prevalence of GORD in older age groups and the aging of the population.^2^ As the UK population continues to age in the coming years, GORD is likely to become even more prevalent, with more patients seeking access to innovative, effective treatment. Given that GORD patients with frequent and/or severe symptoms, who are perhaps most likely to seek surgical treatment, experience a substantial impairment of both health-related quality of life and work productivity,^30^ the broader societal benefits of a novel effective treatment for the condition are likely to be substantial.

With regard to uncertainties and limitations of the current analysis, it should be noted that the results of the model are largely driven by the market share inputs. As these are associated with substantial uncertainty, and the real-world uptake of RefluxStop™ is difficult to predict, scenario analyses were conducted to assess the effect of halving or doubling the rates of RefluxStop™ uptake. In both of these scenarios, the budget impact of introducing the treatment remained small and manageable. Other inputs that are likely to substantially impact the model results are the size of the eligible patient population, and therefore the prevalence and incidence of GORD, the proportion of patients on PPIs who have not had prior surgical treatment for GORD, and the proportion of those willing to undergo surgery and eligible for it. An additional source of uncertainty around the results of the current analysis arises from the estimates of the total costs, which were based on a previously published cost-effectiveness model of RefluxStop™.^10^ Although there was uncertainty around some of the inputs of that model, it predominantly used well-established, standard cost sources for England and its results proved robust to rigorous deterministic and probabilistic sensitivity analyses,^10^ increasing confidence in using the model as a source of cost data for the current analysis.

## CONCLUSION

Introducing RefluxStop™ as a treatment option for patients with GORD treated within NHS England and Wales may be associated with clinical benefits at the expense of a marginal budget impact on the NHS over a 5-year time horizon, assuming the model is reflective of real-world outcomes. Considering the likely increase in both medical costs and the wider societal impact of GORD as the UK population continues to age, an effective and cost-effective treatment option for GORD patients that is also economically acceptable in the short term is likely to provide substantial benefits to patients, the NHS, and the broader society.

### Disclosures

S.M., S.H., and L.G. work for a consulting company that works for a range of medical devices companies, including Implantica. They have no other interests to declare. E.G., P.G., and A.R.A have no interest to declare.

## Supplementary Material

Supplementary Online Material
