# Supplementary material for: Budget Impact of RefluxStop™ as a Treatment for Patients with Refractory Gastro-oesophageal Reflux Disease in the United Kingdom
Source: J Health Econ Outcomes Res. 2024 Jan 11;11(1):1–7. doi: 10.36469/001c.90924 (PMC10787539; doi:10.36469/001c.90924)

Journal of Health Economics and Outcomes Research

Online Supplementary Material

Budget Impact of RefluxStop™ as a Treatment for Patients with Refractory Gastro-oesophageal Reflux Disease in the United Kingdom. *JHEOR*. 2024;11(1):1-7. doi:10.36469/jheor.2024.90924

Figure S1: Schematic of the RefluxStop™ Budget Impact Model

This supplementary material has been provided by the authors to give readers additional information about their work.

This is an open-access article distributed under the terms of the Creative Commons Attribution 4.0 International License (CCBY-4.0). View this license's legal deed at http://creativecommons.org/licenses/by/4.0 and legal code at http://creativecommons.org/licenses/by/4.0/legalcode for more information.

Figure S1. Schematic of the RefluxStop™ Budget Impact Model

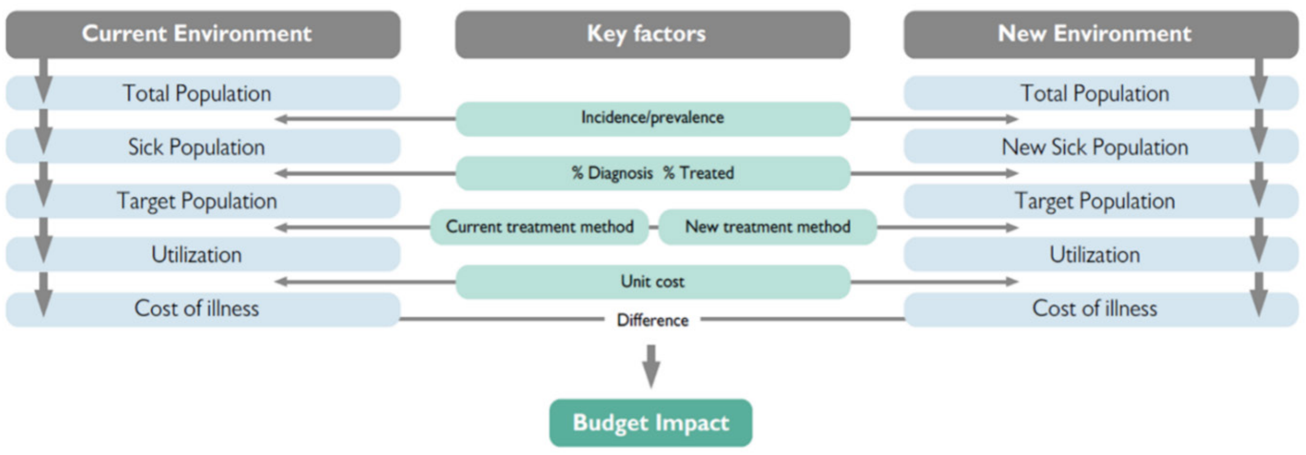

Supplement: Supplementary Online Material [file jheor_2024_11_1_90924_192126.pdf]
